# Navigating Challenging Left Ventricular Lead Placements for Cardiac Resynchronization Therapy

**DOI:** 10.19102/icrm.2020.110505

**Published:** 2020-05-15

**Authors:** Naga Venkata K. Pothineni, Gregory E. Supple

**Affiliations:** ^1^Hospital of the University of Pennsylvania, Philadelphia, PA, USA; ^2^University of Pennsylvania Perelman School of Medicine, Philadelphia, PA, USA

**Keywords:** Cardiac resynchronization therapy, left ventricle, lead implantation

## Abstract

Cardiac resynchronization therapy (CRT) is a mainstay in the management of heart failure patients with electrical dyssynchrony. Left ventricular (LV) lead positioning remains an important variable that predicts the response to CRT. Anatomical and technical challenges can hinder optimal LV lead placement using traditional lead implantation approaches. Knowledge of normal anatomical variants and common anomalies is essential for successful LV lead implants. With advancements in tools and techniques for LV lead delivery, the implanting electrophysiologist can target the optimal LV pacing site, rather than accepting a suboptimal location that is less likely to provide clinical benefit. In this review, we discuss various challenges to achieving optimal LV lead implantation and present strategies to overcome them.

## Introduction

Cardiac resynchronization therapy (CRT) is a well-established treatment strategy for patients with heart failure with reduced ejection fraction and evidence of interventricular dyssynchrony.^1^ An essential component of optimal CRT delivery is the placement of a left ventricular (LV) pacing lead through the epicardial venous system to recruit areas of delayed myocardial activation and restore synchrony. Despite preclinical and clinical studies supporting that CRT provides hemodynamic and clinical benefits, a significant proportion of patients receiving CRT remain nonresponders.^2,3^ Among factors responsible for CRT nonresponse, suboptimal LV lead positioning represents an important technical component.^4^ Anatomical limitations imposed by the coronary venous system pose significant challenges to optimal LV lead positioning. A common bailout strategy is the surgical placement of LV epicardial leads. However, LV epicardial lead placement is associated with increased rates of complications such as renal insufficiency and infections.^5^ With the advancements made in tools and techniques for LV lead delivery, the implanting electrophysiologist now has a variety of approaches to adopt when encountering challenging LV lead implants to obtain the maximal clinical benefit. In this review, we discuss various challenges to achieving optimal LV lead implantation and explain strategies to overcome them.

## Coronary sinus clinical anatomy for left ventricular lead placement

The coronary sinus (CS) is the main vein of the venous system of the heart and runs posteriorly in the atrioventricular (AV) groove. The great cardiac vein in the anterior aspect of the AV groove joins with the main posterolateral vein to form the CS, which then drains into the right atrium. The CS is also met by other tributaries such as the middle cardiac vein (MCV) and small cardiac vein. Further, the great cardiac vein is united with the anterior interventricular vein in the anterior interventricular septum. As with other venous systems, the CS has valves. The ostium of the CS in the right atrium is guarded by the Thebesian valve, which can have a variable structure, sometimes completely covering the ostium and posing a challenge in CS cannulation. The valve of Vieussens demarcates the junction between the CS and the great cardiac vein and represents the termination of the main CS.^6^ The CS ostium is located in the right atrium in the posterior aspect of the interatrial septum. The Eustachian ridge lies in the anterior aspect of the CS ostium. In some cases, where the Eustachian ridge is prominent, there is often an accompanying sub-Eustachian pouch just posterior to the tricuspid annulus, which can provoke problems during CS cannulation.^7^ An additional vein, the vein of Marshall, is usually present and represents the embryological remnant of the left superior vena cava (SVC). In some cases of persistent left SVC or CS ostial atresia, the vein of Marshall is of a large caliber and may be cannulated for lead placement. The anterior interventricular vein is the largest of all the CS tributaries and runs in the anterior interventricular groove parallel to the left anterior descending artery. At the junction of the anterior interventricular vein with the GCV, anterolateral branches can sometimes be found and adopted for LV lead cannulation. The posterolateral veins drain the majority of the lateral wall of the LV and represent ideal targets for LV lead placement as they represent the site of latest activation during RV pacing and serve as the best site for resynchronization. The MCV is the most proximal tributary of the CS and runs in the posterior interventricular groove. Although not an ideal site for LV lead placement, the lateral branches of the MCV can be selected for LV lead implant, and cannulation of the MCV may also aid in the placement of LV leads in other posterolateral branches using advanced techniques.

### Challenge 1: stenotic or occluded thoracic venous system

**Figure 1:**
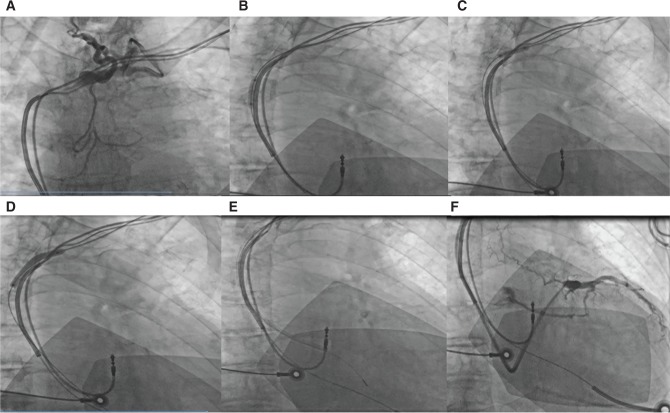
Subclavian venoplasty: **A:** Occluded left subclavian vein. **B and C:** A 6-mm × 4-cm balloon was passed along a guidewire over the stenotic segment for serial inflations (distal then proximal) along the area of stenosis. **D:** Longer and larger balloon dilation. **E and F:** Eventual passage of the sheath for CS cannulation.

One of the common challenges encountered during the implantation of cardiac implantable electronic devices (CIEDs) is stenosis and occlusion of the venous system in the thorax. This is more relevant during an upgrade procedure with the addition of LV leads in patients with preexisting device systems. It is estimated that 10% to 30% of patients with existing CIEDs have stenosis or occlusion of the axillary and subclavian venous system.^8^ This finding often leads to abandonment of the procedure, implantation from the contralateral side and tunneling of the lead to the existing pocket, or referral for surgical epicardial LV lead implantation. All these approaches are more aggressive and expose the patient to higher risks for complications. In contrast, a simpler and more straightforward approach is venoplasty. This approach involves obtaining access proximal to the site of occlusion and placement of a 5-French sheath in the open part of the vein. If the vein is stenotic, a soft-tipped guidewire is then used to traverse the occlusion. If, however, the venous system is completely occluded, often with a combination of hydrophilic wires and small steerable vein selectors, the stenosis can be effectively crossed while keeping the wire in the true lumen of the vein. A variety of angioplasty wires (0.014-inch, 0.018-inch, and 0.035 inch) may be used often in a steerable vein selector; however we have found that a hydrophilic wire such as the Glidewire^®^ (Terumo, Tokyo, Japan) may be the best tool for crossing an occlusion. Often, a microcatheter is needed to provide support to the wire to cross the occlusion.^9^ Once across the occlusion, a balloon (6 mm × 4 cm) is loaded over the wire. It is important to initiate balloon dilation in the most distal part of the stenosis and conduct serial dilations in a distal to proximal fashion. The profile of the balloon increases after the first dilation—a phenomenon called winging—and may impede further advancement if inflated in the proximal aspect of the stenosis first. If the obstruction is severe and accommodates only a 0.014-in (0.04-cm) angioplasty wire, then initial dilations with a 3-mm coronary balloon may aid in exchange of the wire to accommodate larger and stiffer wires (such as the Glidewire^®^ from Terumo, Tokyo, Japan). This technique, although less frequently employed, appears to be very safe and has a very low risk of major complications.^10^ Areas of venous occlusion are usually heavily laden with fibrous tissue, which minimizes the chances of venous wall injury and rupture. In cases of severe obstruction precluding access to the central venous system, lead extraction followed by reimplantation is probably a better alternative to contralateral lead implantation to reduce the overall lead burden and associated complications. The steps involved in subclavian venoplasty are outlined in **Figure 1**.

### Challenge 2: difficult coronary sinus access

Multiple approaches to CS cannulation are used and are operator-dependent. Techniques employing an electrophysiology catheter, soft-tipped guidewires, or soft-tip sheaths with contrast injection during CS cannulation have been well-described.^11^ Challenges to cannulating the CS include a dilated right atrial cavity, prominent Thebesian valve, narrow CS body, and areas of focal stenosis in the CS. A routine approach of entering the right ventricle and then retracting the CS sheath from the para-Hisian region with a counterclockwise torque (orienting the tip of the sheath in a septal and inferior direction) generally helps to cannulate the CS ostium and prevent the sheath getting caught in a Thebesian valve. Occasionally, one encounters a CS ostium that is difficult to locate using this approach. Considering the lucency of the fat pad, a right anterior oblique projection can help to identify the general fluoroscopic location of the CS ostium. In rare cases, a venous-phase coronary angiogram can delineate the venous anatomy of the left ventricle and act as a fluoroscopic guide to CS cannulation. This can also help identify unusual cases of CS ostial atresia.^12^

Once the CS ostium is cannulated, further challenges with advancing the sheath into the CS body can present. This may be secondary to tortuosity or stenosis of the CS body and can be circumvented using tools that provide additional rail support such as inner sheaths or using an anchor balloon technique. One maneuver is to advance a vein selector over an angled 0.035-in (0.09-cm) Glidewire^®^ (Terumo, Tokyo, Japan) deep into the CS. Keeping the vein selector deep in the CS, the Glidewire^®^ (Terumo, Tokyo, Japan) is replaced with a 0.035-in (0.09-cm)-diameter, 180-cm-long J-tip Amplatz Extra Stiff™ wire (Boston Scientific, Natick, MA, USA) that acts as a stronger rail to advance the CS sheath across areas of narrowing in the CS body. Following this, the application of telescoping sheaths to selectively cannulate smaller branches of the CS can provide additional stability to the CS outer sheath during lead positioning and has been shown to reduce procedural time.^13^

A second technique available for difficult CS cannulation is the anchor balloon technique. With this approach, the ostium of the CS is engaged with a small-caliber sheath through which a 0.014-in (0.04-cm) angioplasty wire is advanced into the CS body. Then, a 3-mm compliant coronary balloon is advanced over the angioplasty wire deep into the CS and preferably into one of the tributaries of the CS, such as the anterior interventricular vein. The balloon is then inflated to create an anchor.^14^ With gentle traction on the balloon, the CS sheath is then advanced into the body of the CS and the angioplasty wire is exchanged to a 0.035-in (0.09-cm) Amplatz Extra Stiff™ wire (Boston Scientific, Natick, MA, USA) for additional rail support. With these techniques, most commercially available CS outer sheaths can be successfully advanced into the mid-CS to distal CS, enabling LV lead advancement.

### Challenge 3: tortuous and stenotic venous branches

**Figure 2:**
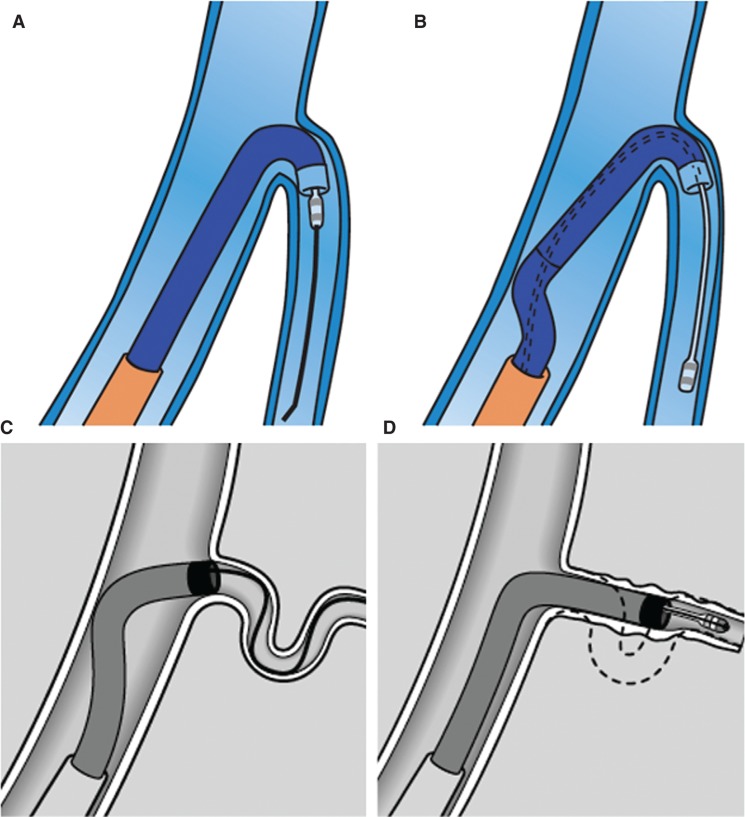
**A and B:** Using a renal inner sheath to provide support from the far wall of the CS to push the lead. **C and D:** Advancing the sheath over the wire to straighten out the tortuosity and advance the lead.

**Figure 3:**
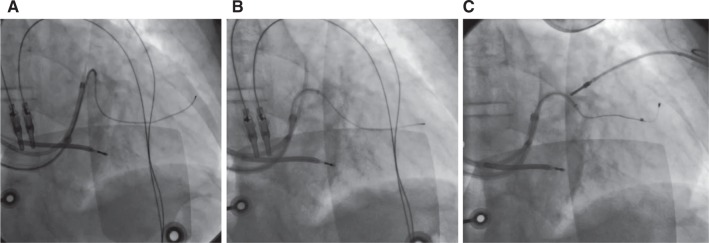
**A:** Acute take-off of a venous branch straightened by advancement of **B:** the inner sheath, thus **C:** enabling progression of the pacing lead.

**Figure 4:**
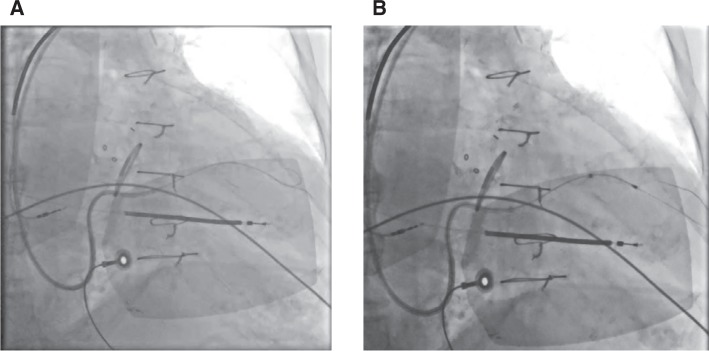
Buddy wire technique to enhance support for sheath advancement into **A:** the venous branch of interest, thus **B:** enabling progression of the pacing lead.

**Figure 5:**
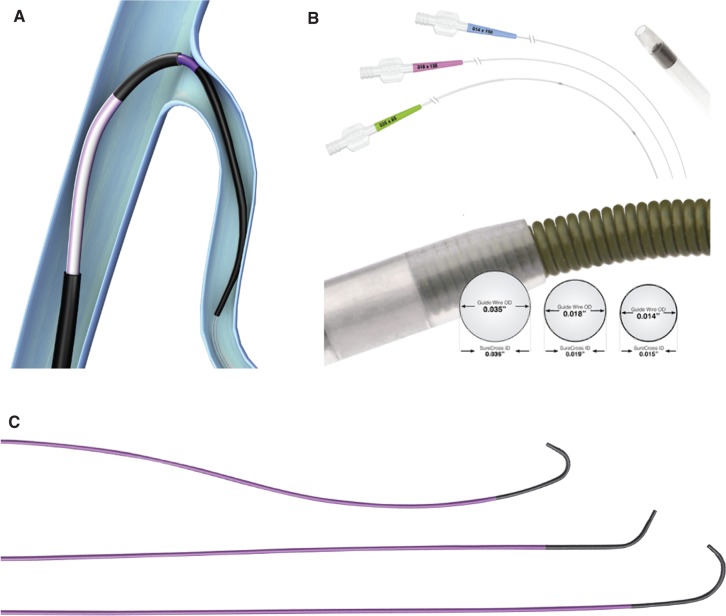
**A:** Schematic showing use of a lateral vein introducer and a vein selector for selective cannulation of a target branch. **B:** Profiles of various guidewires and microcatheters. **C:** Different shapes of Worley vein selectors (ie, standard, vertebral, and hook).

**Figure 6:**
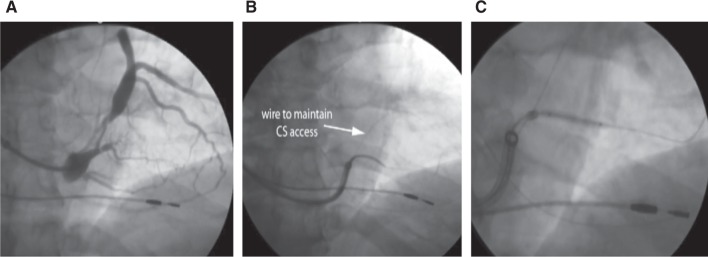
**A:** Small-caliber and stenotic venous branch of interest. **B:** Selective cannulation of the branch with an inner sheath with a second wire in the CS to maintain access. **C:** Coronary venoplasty with an angioplasty balloon to enable wire advancement and eventual lead placement. Images courtesy of Seth Worley.

Once the CS sheaths are in place, a good-quality occlusive venogram helps the operator to identify venous branches of interest for the placement of LV pacing leads. A typical LV lead implant involves a technique of advancing the pacing lead over an angioplasty wire that is parked distally in a target branch. However, a frequent challenge encountered is small-caliber veins that do not accommodate the LV lead or tortuosity of the branches with acute angulations that impede lead advancement. This is a common cause for choosing a less tortuous, albeit suboptimal branch for LV lead placement, which significantly impacts the clinical benefit of biventricular pacing. Negating venous tortuosity by using telescoping sheaths to selectively cannulate venous branches can help straighten the path to LV lead advancement **(Figures 2 and 3)**. Geometrically, the more acute the angle of take-off of a branch, the higher the chances of the forward force being transmitted back and causing dislodgement of the CS apparatus. Double-wiring the branch **(Figure 4)** or using additional support with stiffer guidewires can assist with selective cannulation of the venous branches with an inner sheath or vein selectors.^15^ **Figure 5** illustrates common tools that are useful in cannulating tortuous branches and stepping up sheath support. A less commonly used technique in patients with stenosis of the venous branches in coronary venoplasty involves using a 3-mm angioplasty balloon.^16^ This has been reported to have a low incidence of adverse events and can help facilitate lead advancement in patients with very focal stenosis of the target vein **(Figure 6)**.

### Challenge 4: tiny venous branches

Target veins in optimal locations for LV lead placement are sometimes small and do not appear likely to accommodate a pacing lead. Instead of choosing a suboptimal larger branch in these situations, adopting the snare technique and taking advantage of natural bridging collateral veins enables the operator to overcome this challenge and implant the lead in the branch of interest that is most likely to provide clinical benefit.^17^ Depending on the direction in which the LV lead is advanced, there are two types of snare techniques that can be applied.

**Figure 7:**
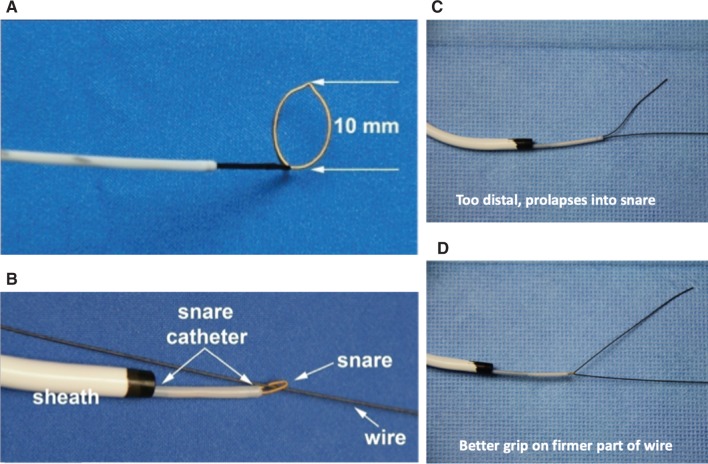
**A:** Structure of a 4-French snare catheter system. **B:** A guidewire was passed through the snare and the snare loop closed. **C:** Snaring a wire distally in the floppy part caused the wire to prolapse. **D:** Snaring on the firmer part of the wire achieves a better grip and support for a rail.

**Figure 8:**
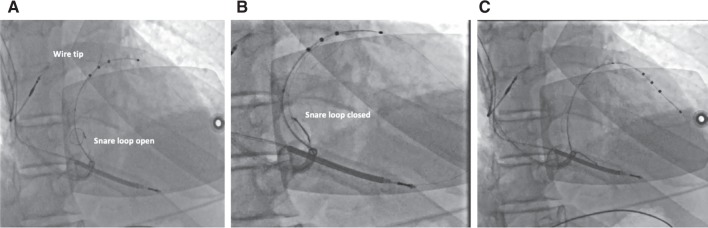
Orthodromic snare technique. **A:** The wire is advanced through collaterals and back into the body of the CS. The snare is advanced adjacent to the lead into the body of the CS. **B:** The wire is snared about 10 cm to 15 cm from the tip. **C:** The lead is advanced to the desired position with gentle traction and countertraction.

In the orthodromic snare technique, a vein selector is advanced over a polymer-tip floppy wire through the CS outer sheath and used for selective cannulation of the branch of interest. The wire is then advanced as distally as possible to provide an additional rail by which to progress the vein selector into the branch. If the first wire is met with resistance and further steerability is lost due to a deformed tip, it can be left in the branch and a second new wire can be advanced to be the primary wire. A second option is to exchange the wire though a microcatheter without losing the ground already made with the first wire. With a vein selector in a branch, the wire is manipulated to attempt to advance it through collateral bridging veins such that it can be retrogradely progressed through another branch back into the body of the CS. In our experience, the Choice PT floppy wire (Boston Scientific, Natick, MA, USA) is best suited to finding and traversing through collateral veins, and involving a vein selector can help steer through different potential collaterals. A selective venogram of the target branch in question can also be performed via the vein selector to more clearly identify potential collaterals that can be crossed. Once the wire has been successfully advanced through collaterals with the tip back in a CS, a 4-French snare is then advanced (either through a single large sheath such as the Worley sheath if it was used or through a second venous access sheath) with careful attention given to the position of the vein selector so as not to disengage the venous branch of interest. Once the snare is positioned in the CS body, the wire that was advanced through the collaterals is fed through the snare loop and the loop is tightened. It is important to close the snare loop on the stiff part of the wire and not the distal floppy portion to ensure a good grip and to maintain traction **(Figure 7)**. Once the snare is tightened, the snare apparatus provides excellent distal support for the inner sheath and aids lead advancement, which can now be pulled into location using distal traction from the snare **(Figure 8)**. In fact, the CS outer sheath can be completely disengaged from the CS ostium at this point if needed.^18^

**Figure 9:**
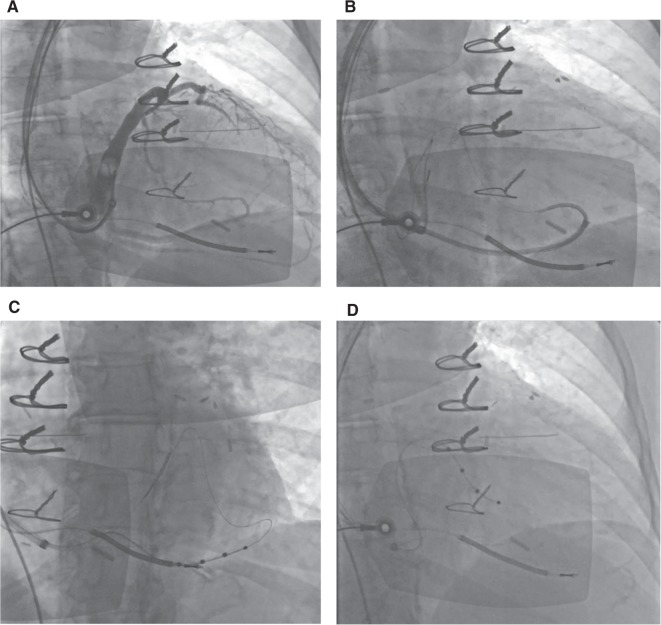
Antidromic snare technique. **A:** Venogram showing extremely tortuous and small-caliber target vein. **B:** The vein selector was advanced retrogradely. **C:** The lead was advanced to this branch with poor location and parameters so was removed. **D:** The distal wire was snared and pulled all the way back out, and the lead was advanced retrogradely into the target, a tortuous superior branch.

In cases where the wire cannot be advanced antegradely through the target venous branch due to extreme angulation, the antidromic snare technique can be employed. In this technique, the CS is cannulated with a 9-French outer sheath through which a vein selector is advanced into the CS over a support wire. The 0.035-in (0.09-cm) wire is then replaced with an Amplatz Extra Stiff™ wire (Boston Scientific, Natick, MA, USA) to provide additional support and to retain CS access. The vein selector is then removed and advanced over a wire to selectively cannulate a vein not intended as a target (therefore, the MCV or anterior interventricular vein may be used). A good initial venogram or subsequent selective venography from different CS branches may identify suitable branches that have collateralization with the target vessel. The vein selector is then advanced down the alternative branch and a 300-cm Choice PT floppy wire is maneuvered through the collateral network into the target vein retrogradely and back into the body of the CS. The snare catheter is then inserted into the CS. The wire that was retrogradely advanced through the target branch is then fed through the loop in the body of the CS where it is snared and secured. The vein selector is then removed and a microcatheter is advanced over the retrograde part of the wire, through the collaterals and back into the CS. A 300-cm wire is necessary for this maneuver, as the wire is then pulled back through the microcatheter until the tip is externalized in the pocket; with this, both ends of the wire are now available for use in the pocket for antegrade or retrograde lead advancement. The snare is then removed and the pacing lead is loaded retrogradely onto the externalized wire. Gentle traction and countertraction of the wire during lead advancement enables the pacing lead to be positioned in the branch of interest **(Figure 9)**.

### Challenge 5: right-sided implants

**Figure 10:**
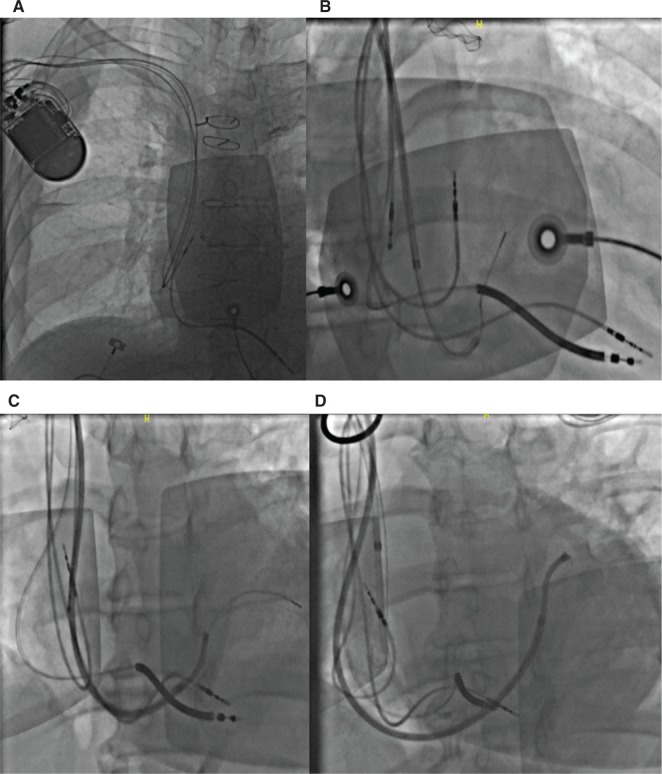
Right-sided CS cannulation. **A:** Chest X-ray showing acute angulation of the right subclavian–SVC junction. **B:** The outer sheath has a relatively straight course during CS cannulation. **C:** The outer sheath running along the lateral right atrial wall to facilitate advancement of the wire and the inner sheath into the CS. **D:** Optimal CS sheath position for successful lead delivery.

Most traditional CRT systems are implanted on the left side. However, venous occlusions, device site infection, the presence of infusion ports, arteriovenous fistulas for hemodialysis, and patient preferences can often necessitate right-sided implants. LV lead implantation from the right subclavian venous system presents anatomical and technical challenges, the major one being a difference in ergonomics, as catheter manipulation tends to be opposite the usual practice. Making changes to the table setup so that the operator faces the feet of the patient during right-sided implants has been reported to replicate catheter manipulation similar to left-sided implants. Anatomically, the right subclavian vein has a shorter and more vertical course before joining the SVC. This variation leads to two main differences in CS cannulation. First, a large portion of the sheaths used are typically outside the body during right-sided implants and would need extra attention to prevent contamination. Second, sheaths advanced through the right side have a reverse curve as they traverse into the SVC, which limits the ability to transmit torque gradually. Finally, upon entering the right atrium, the sheath takes a straighter and more septal course with no natural curve to provide support against the lateral wall **(Figure 10)**. To help overcome these challenges, cannulating the CS with a telescoping system, where an outer sheath is parked at the SVC-RA junction and an inner sheath is laid along the lateral wall of the right atrium, will often aid in reaching the CS. The outer sheath at the SVC-RA junction can be torqued to direct the inner sheath to lie along the lateral atrial wall, which then provides enough support to rail the outer sheath over it once the CS is cannulated. The use of inner sheaths or guides with large secondary curves often facilitates successful CS cannulation from the right side by directing the tip of the sheath higher on the annulus. Once the CS is cannulated, LV lead delivery is similar to that with left-sided implants.

A frequently encountered challenge with right-sided implants is lead dislodgment while removing the outer sheath. This happens due to the tendency of the outer sheath to flop toward the right atrial lateral wall once disengaged from the CS in addition to the acute angle at the subclavian–SVC junction that needs to be overcome during slitting of the sheath. A well-anchored lead (using some of the techniques discussed previously) may prevent dislodgement, while leaving an extra-stiff wire adjacent to the lead in the CS body while withdrawing the outer sheath may provide a smoother rail and prevent the sheath from whipping out of the CS.

### Challenge 6: persistent left superior vena cava

**Figure 11:**
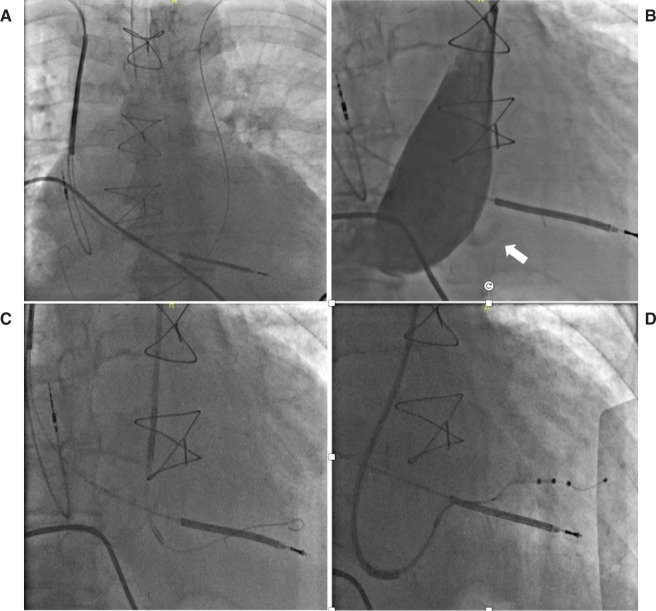
Lead implantation via PLSVC. **A:** Guidewire from the left axillary vein access entering the PLSVC. **B:** Venogram showing a massively dilated CS with the hint of a posterolateral branch (arrow). **C:** Selective cannulation of the branch using an inner sheath over the wire. **D:** Ultimate lead position after using outer and inner sheaths to provide support in the dilated CS body.

Persistent left SVC (PLSVC) is a congenital developmental abnormality of the sinus venosus with an incidence of approximately one in 200 patients undergoing CIED implant.^19^ Two variants include a double SVC (right and left SVCs, with or without an innominate vein connecting the two) or a single left-sided SVC (without a right SVC) that drains into the CS. The former variant is more common, and implantation of LV leads in such cases can be achieved with good success rates. Although the presence of a dilated CS makes CS cannulation easier, several technical challenges are encountered that hinder appropriate LV lead positioning. As the CS is often massively dilated, a completely occlusive venogram is often not possible. However, advancement of the balloon tip beyond the Vieussens valve, distal to the insertion of the PLSVC, often enables a good-quality occlusive venogram as the vein caliber of the venous system at this position is usually normal. Using a vein selector to guide the wire to enter the venous system beyond the Vieussens valve, which can then be selectively cannulated for lead advancement, often results in success. In cases of absent right-sided SVC, the only route by which to implant pacing leads is through the PLSVC. Although this provides a direct route to the CS, LV lead implantation through a PLSVC is extremely challenging, as an approximately 180° turn has to be maneuvered to enter the venous system beyond the Vieussens valve. In these cases, posterolateral branches that arise in the proximal portions of the CS often present viable targets due to a straighter course **(Figure 11)**. However, once an LV lead is placed, the lack of space for adequate lead slack and high blood flow down the PLSVC into the CS can increase the risk of lead dislodgement. Again, using some of the techniques described above, such as snaring a lead that is well-anchored, may help operators to overcome this challenge. For de novo implants, if a PLSVC is detected during venous access and there is no direct communication to the normal SVC from the L side, we recommend evaluating the right side for the presence of a right SVC and, if present, consider pursuing a right-sided implant to improve the ease of LV lead placement and reduce the risk of lead dislodgement. For patients with existing left-sided systems who need upgrades with the addition of an LV lead and who have a PLSVC, implanting the LV lead from the right side and tunneling to the left-sided pocket can be considered.

## Conclusion

CRT is a mainstay in the management of heart failure patients with electrical dyssynchrony. LV lead positioning remains an important variable that predicts response to CRT. Anatomical and technical challenges can hinder optimal LV lead placement using traditional lead implantation approaches. Knowledge of normal anatomical variants and common anomalies is essential for successful LV lead implants. An understanding of the available tools and techniques to facilitate LV lead delivery can assist in achieving successful outcomes when encountered in a challenging case. The optimal LV pacing site can be targeted and achieved with these skills, rather than accepting a suboptimal location that is less likely to provide clinical benefit. While new frontiers in cardiac resynchronization such as conduction system pacing and LV endocardial pacing are being actively evaluated and hold promise to improve patient outcomes, CRT with optimal LV lead placement remains the therapy of choice backed by randomized trial data and will certainly continue to hold an important place in the field of CRT. Attention to detail and perseverance are crucial to achieving success in difficult cases of CRT.
